# White Adipose Tissue Expansion in Multiple Symmetric Lipomatosis Is Associated with Upregulation of CK2, AKT and ERK1/2

**DOI:** 10.3390/ijms21217933

**Published:** 2020-10-26

**Authors:** Marta Sanna, Christian Borgo, Chiara Compagnin, Francesca Favaretto, Vincenzo Vindigni, Mariangela Trento, Silvia Bettini, Alessandra Comin, Anna Belligoli, Massimo Rugge, Franco Bassetto, Arianna Donella-Deana, Roberto Vettor, Luca Busetto, Gabriella Milan

**Affiliations:** 1 Endocrine-Metabolic Laboratory, Department of Medicine, Internal Medicine 3, University of Padua, 35128 Padua, Italy; sanna_marta@yahoo.it (M.S.); chiara.compagnin@unipd.it (C.C.); francesca.favaretto@unipd.it (F.F.); d.ssa.silvia.bettini@gmail.com (S.B.); alessandra.comin@tin.it (A.C.); anna.belligoli@gmail.com (A.B.); roberto.vettor@unipd.it (R.V.); luca.busetto@unipd.it (L.B.); 2 Department of Biomedical Sciences, University of Padua, 35131 Padua, Italy; christian.borgo@unipd.it (C.B.); arianna.donella@unipd.it (A.D.-D.); 3 Clinic of Plastic and Reconstructive Surgery, Department of Neurosciences, University of Padua, 35128 Padua, Italy; vincenzo.vindigni@unipd.it (V.V.); franco.bassetto@unipd.it (F.B.); 4 Surgical Pathology and Cytopathology Unit, Department of Medicine, University of Padua, 35121 Padua, Italy; mariangela.trento@hotmail.it (M.T.); massimo.rugge@unipd.it (M.R.)

**Keywords:** multiple symmetric lipomatosis, white, brown and beige adipocytes, adipose stem cells, CK2, AKT, ERK1/2

## Abstract

Multiple symmetric lipomatosis (MSL) is a rare disorder characterized by overgrowing lipomatous tissue (LT) in the subcutaneous adipose tissue (SAT). What LT is and how it expands are not completely understood; previous data suggested that it could derive from brown AT precursors. In six MSL type I patients, we compared LT morphology by histological and immunohistochemistry (IHC) analysis, gene expression, by qPCR, kinase activity, by Western Blot and in vitro assay to paired-control SAT using AT from patients with pheochromocytoma as a human browning reference. In the stromal vascular fraction (SVF), we quantified adipose stem cells (ASCs) by flow cytometry, the proliferation rate, white and beige adipogenic potential and clonogenicity and adipogenicity by a limiting dilution assay. LT displayed white AT morphology and expression pattern and did not show increased levels of the brown-specific marker UCP1. In LT, we evidenced AKT, CK2 and ERK1/2 hyperactivation. LT-SVF contained increased ASCs, proliferated faster, sprouted clones and differentiated into adipocytes better than the control, displaying enhanced white adipogenic potential but not increased browning compared to SAT. In conclusion, LT is a white AT depot expanding by hyperplasia through increased stemness and enhanced white adipogenesis upregulating AKT, CK2 and ERK1/2, which could represent new targets to counteract MSL.

## 1. Introduction

Multiple symmetric lipomatosis (MSL, #OMIM 151800) is a rare disorder characterized by enlarging, painless, symmetric and unencapsulated lipomas developing in the subcutaneous adipose tissue (SAT). Two types of MSL patients are clinically described [1,2]: type I patients present well-circumscribed, grossly round lipomas protruding from the body surface and a substantially normal body mass index (BMI), while type II patients are characterized by lipomatous tissue (LT) widespread in SAT, mimicking an obesity-like phenotype. Fat masses are present most frequently at the neck (83.3% of the male patients), dorsal area (55%) and upper limbs (54.1%); distal segments of the arms and legs are invariably spared [3]. MSL is more prevalent in men (M:F ratio about 6:1), and type II is more frequent in female patients. MSL is associated with ethanol intake, which plays a still unclear role in the pathophysiology of this disease [2]. Although familial cases of MSL have been described, the genetic alterations causing the disease are still unknown. Both autosomic dominant and recessive inheritance have been hypothesized and variations in mitochondrial DNA (mtDNA) or *MFNT2*, a gene involved in mitochondrial fusion associated with other syndromes or complex diseases, were identified in some MSL cases [4,5,6,7,8].

The mechanisms involved in LT formation are not yet completely understood, even if its anatomical localization and previous tissue analyses indicate its possible origin from brown AT (BAT) precursors [9,10]. Some authors suggest that LT fails to completely differentiate into mature BAT due to mitochondrial dysfunction [11,12] and/or defective noradrenergic regulation [13].

Phenotype characterization of *PTEN*^myf5cKO^ mouse, a selective knockout of phosphatase and tensin homolog (*PTEN*) in Myf5^+^ lineage, opened new insight into the molecular mechanisms underlying MSL pathology [14]. This mouse model recapitulated the fat mass distribution of MSL with an enlargement of interscapular white AT (WAT), reduction of inguinal and epidydimal depots and limb muscle atrophy. Since PTEN acts as a potent inhibitor of the insulin cascade, these data suggest a possible role of insulin signaling hyperactivation in MSL pathogenesis, as observed in other types of AT overgrowth [15].

Moreover, miRNAs promoting in vitro adipogenesis (miR-125a-3p and miR-483-5p) were reported to be upregulated in MSL, leading to the inhibition of the RhoA/ROCK/ERK1/2 pathway [16]. Lastly, a downregulation of Calcyphosin-like (*CAPSL*) has been recently suggested to be involved in both adipogenesis and autophagy of MSL patient LT [17].

The present work provides an extensive morphological and functional characterization of LT and LT-derived adipose stem cells (ASCs) in six MSL type I patients. Healthy SAT samples harvested from the same patients were used as controls. We investigated the activation state of CK2, AKT and ERK1/2, protein kinases involved in insulin signaling and/or proliferation pathways, in LT samples with different anatomical localization.

## 2. Results

### 2.1. Morphological and Gene Expression Analyses Reveal White Features of LT

**Figure 1:**
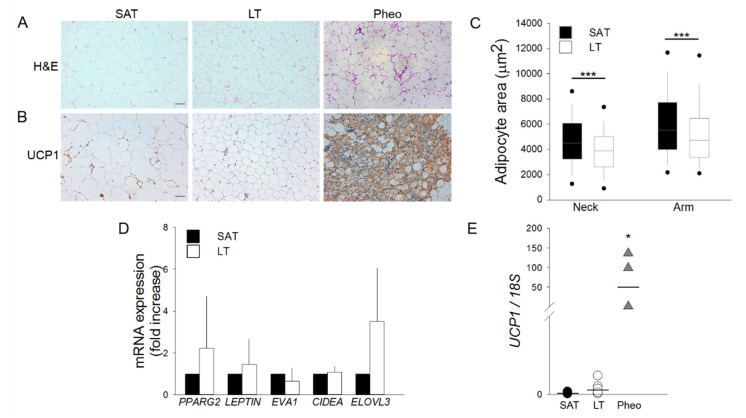
Lipomatous tissue (LT) displays a white morphology. (**A**,**B**) Representative photomicrographs of sections of LT, subcutaneous adipose tissue (SAT) of multiple symmetric lipomatosis (MSL) patients and perirenal adipose tissue (AT) of patients affected by pheochromocytoma (Pheo) stained with (**A**) hematoxylin and eosin (H&E) and (**B**) anti-UCP1 antibody. Scale bar: 100 µm. (**C**) Measurements of adipocyte area (µm^2^) in the LT (white boxes) harvested from neck (*n* = 2 patients) and arm (*n* = 3 patients) and paired SAT (black boxes). Data are representedk as box plot graphs with medians (lines), lowest and highest values (whiskers) and 5th and 95th percentiles (black circles). *** *p* < 0.001 LT vs. SAT, Mann-Whitney test. (**D**) *PPARG2*, *LEPTIN*, *EVA1*, *CIDEA* and *ELOVL3* mRNA levels in LT (white bars, *n* = 6 samples from 5 patients) reported as fold increase with respect to SAT (black bars, *n* = 5 patients). Data are reported as mean ± SD. (**E**) *UCP1* mRNA expression quantified in SAT (black circles, *n* = 5 patients), LT (white circles, *n* = 6 samples from 5 patients) and Pheo (grey triangle, *n* = 4 patients) normalized to *18S* rRNA content. Data (ratio of arbitrary units) are reported as single values of each subject and medians (solid lines). * *p* < 0.05 Pheo vs. SAT and Pheo vs. LT, Mann-Whitney test.

SAT and LT samples from MLS patients were examined for their morphology by optical microscopy and compared with the perirenal AT of patients affected by pheochromocytoma (Pheo). Since this tissue expresses a high level of the uncoupling protein 1 (*UCP1*) [18,19], it was used as a positive control of human brown/beige AT. Hematoxylin and eosin (H&E) (Figure 1A) and immunohistochemistry (IHC) staining with anti-UCP1 antibody (Figure 1B) demonstrates that both LT and SAT from all the analyzed patients (*n* = 6) contain only mature and univacuolated UCP1-negative adipocytes, while Pheo-derived sections are characterized by islands of small and multilocular UCP1-positive cells that are interspersed with white adipocytes (Figure 1A,B). Moreover, the cell size analysis reveals that LT adipocytes exhibit a significantly smaller area with respect to SAT cells, independently of the lipoma anatomical localization (Figure 1C).

The gene expression profile of white, brown and beige markers was then compared in SAT and LT by real-time PCR (Figure 1D,E). We analyzed the classical white adipose markers Peroxisome proliferator-activated receptor gamma (*PPARG2*) and Leptin (*LEP*) and the following brown/beige specific markers: epithelial V-like antigen 1 (*EVA1*), which discriminates between brown and beige cells, cell death-inducing DFFA-like effector A (*CIDEA*), which is expressed in both brown and beige adipocytes [20], and the elongation of very long chain fatty acid-like 3 (*ELOVL3*), which is a browning marker. Figure 1D shows that the expression of these markers is not significantly different in SAT and LT. Notably, the most specific and reliable brown AT marker, *UCP1*, which is clearly expressed in Pheo adipocytes, is barely detectable in both SAT and LT (SAT vs. LT: 0.0001 vs. 0.0004, *p* = 0.082) (Figure 1E) consistently with the results obtained by IHC analysis (Figure 1B).

### 2.2. Protein Kinases CK2, AKT and ERK1/2 Are Hyperactivated in LT

**Figure 2:**
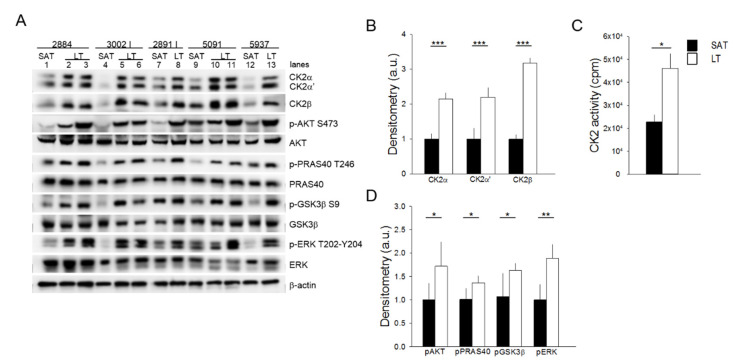
Analysis of CK2, AKT, and ERK1/2 signaling activation. (**A**) Tissue extracts from LT and SAT of 5 patients were analyzed by Western blot with the indicated antibodies. β-actin represents the loading control. 2884, 3002 I, 2891 I, 5091 and 5937 indicate the patient ID numbers; number lanes 1–13 refer to the specific anatomical localization of the samples (see also Table 1 in Materials and Methods): 1, 4, 7, 9 and 12: SAT; 2 and 3: neck LT from two different sites of 2884; 5 and 6: arm and neck LT of 3002 I, respectively; 8: neck LT of 2891 I; 10 and 11: arm and neck LT of 5091, respectively, and 13: arm LT of 5937. (**B**) CK2α, CK2α′ and CK2β immunostained bands shown in (**A**) were quantified by densitometric analysis, and mean values of SAT (black bar) and LT (white bar) extracts ± SD are expressed in arbitrary units (a.u.). *** *p* < 0.001 LT vs. SAT, *t*-test. (**C**) CK2 activity was assayed in SAT (black bar) and LT (white bar) extracts from all 6 patients and expressed as cpm of ^33^Pi transferred to the peptide substrate; data are presented as mean ± SD. * *p* < 0.05 LT vs. SAT, *t*-test. (**D**) The phosphorylation extent of AKT, PRAS40, GSK3 and ERK in SAT (black bar) and LT (white bar) samples from 5 MSL patients was determined by densitometric analysis of the immunostained bands (**A**) and expressed as a ratio of phosphorylated protein/total protein. * *p* < 0.05 and ** *p* < 0.01 LT vs. SAT, *t*-test.

Cellular and molecular mechanisms underlying the pathogenesis of MSL disease remain still unclear. To unravel the potential deregulated pathways for this rare disease, we explored the role of protein kinases that lie at the crossroads of a nutrient-hormonal signaling network that is involved in specific pathological responses, including obesity, diabetes and cancer. An analysis of the protein kinase CK2 in six MSL patients demonstrates that the protein level of both catalytic (CK2α and CK2α′) and regulatory (CK2β) subunits are upregulated in all the examined LT specimens in comparison with the healthy SAT from the same patients (Figure 2A,B) (similar results were obtained with samples from patient 3030, which were analyzed in a different Western blot session). Consistently, CK2 activity tested in vitro toward a specific peptide substrate is about two-fold in LT compared to SAT (Figure 2C). We then examined the protein kinase AKT, whose activation is involved in insulin signaling and cell cancerous transformation [21,22]. Phosphorylation of the regulatory AKT Ser473 residue, which correlates with the kinase activation, is strongly elevated in all LT compared to the paired SAT samples (Figure 2A lanes 1 vs. 2 and 3, 4 vs. 5 and 6, 7 vs. 8, 9 vs. 10 and 11 and 12 vs. 13).

This anomalous activation of AKT is also confirmed by the higher phosphorylation extent of the AKT substrates PRAS40 and GSK3β, involved in the regulation of mTOR signaling and glycogen and lipid synthesis, respectively [23,24]. Lastly, we analyzed the activation state of ERK1/2, a key protein kinase in the regulation of cell proliferation and survival [25]. All the LT samples analyzed contained hyperactivated ERK1/2 compared to the paired SAT, as indicated by the increased phosphorylation of the kinase residues Thr202 and Tyr204 (Figure 2A,D). Hyperactivation of the analyzed protein kinases seems independent of the anatomical localization of lipomas in MSL patients (Figure 2A, lanes 2, 3, 6, 8 and 11 neck lipomas vs. 5, 10 and 13 arm lipomas).

### 2.3. Adipose Stem Cells Are More Abundant in LT Than in SAT

**Figure 3:**
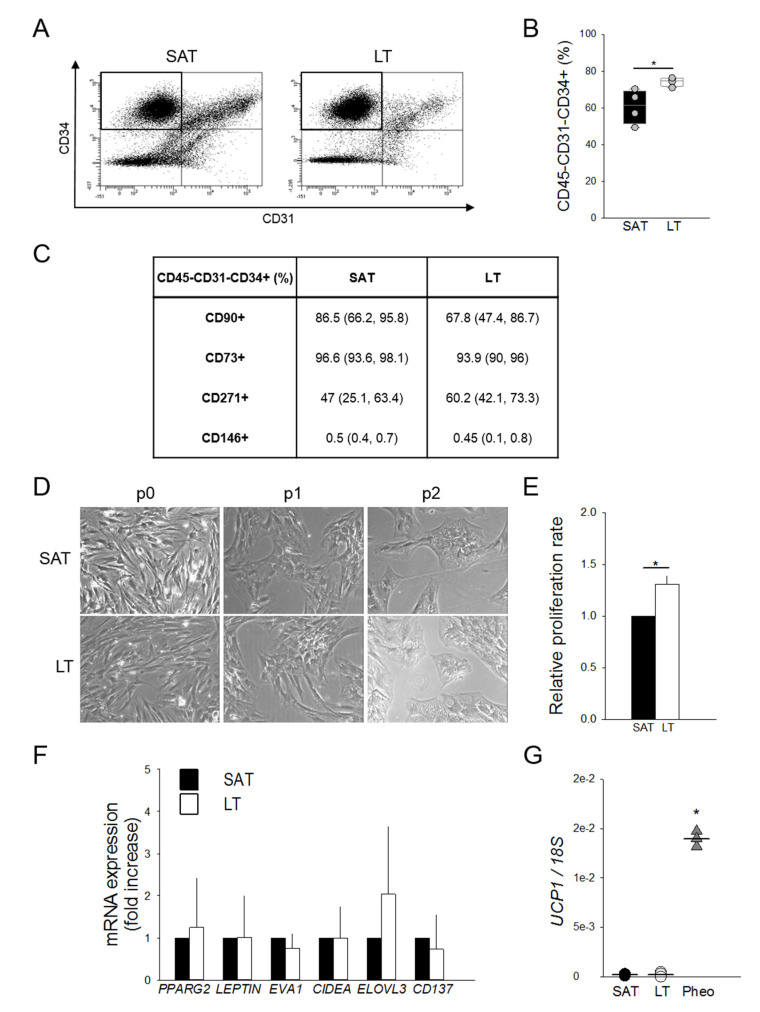
Features and quantification of adipose stem cells (ASCs). (**A**) Representative flow cytometric dot plots of surface markers CD34 vs. CD31, determining the percentage of adipose stem cells (ASCs) as CD45−CD31−CD34+ within stromal vascular fractions (SVFs) freshly isolated from neck LT and paired SAT. (**B**) Quantification of ASCs contained in the SVFs from neck LT (white box) and paired SAT (black box) of 4 patients affected by MSL. The percentage of ASCs are reported as box plot graphs with medians (lines) and single values of each patient (grey circles). * *p* < 0.05 LT vs. SAT, Mann-Whitney test. (**C**) Percentages of CD45−CD31−CD34+ cells expressing CD90, CD73, CD271 and CD146 antigens in LT and paired SAT SVFs of 4 MSL patients. Data are presented as median percentage (25th and 75th percentiles). (**D**) Representative images at optical microscopy (20× magnification) showing SAT- and LT-derived preadipocytes morphology after different culture passages (p0, p1 and p2). (**E**) Proliferation rate (fold increase) of SVF cells from LT (white bar) of 2 MSL patients with respect to paired SAT (black bar), determined using two different experimental methods. Data are reported as mean values ± SD. * *p* < 0.05 LT vs. SAT, *t*-test. (**F**) Gene expression analysis of freshly isolated SVF cells from SAT (black bars) and LT (white bars) of 4 subjects affected by MSL. *PPARG2*, *LEPTIN*, *EVA1*, *CIDEA*, *ELOVL3* and *CD137* mRNA levels, normalized to *18S* rRNA content, are expressed as a fold increase with respect to SAT-derived SVF cells. Data are reported as mean values ± SD. (**G**) *UCP1* mRNA levels in SAT-derived SVF (black circles, *n* = 4 patients), LT SVF (white circles, *n* = 5 samples from 4 patients) and Pheo SVF cells (grey triangles, *n* = 3 patients), normalized to *18S* rRNA content. Data (ratio of arbitrary units) are reported as values of each subjects (circles or triangles) and medians (solid lines). * *p* < 0.05 Pheo vs. LT, Mann-Whitney.

To investigate the hypothesis of a potential hyperplastic growth of MSL pathological tissue, ASCs were ex vivo quantified by flow cytometry as the percentage of CD45−CD31−CD34+ cells in paired LT- and SAT-derived stromal vascular fractions (SVFs) harvested from MSL patients. Interestingly, LT is significantly enriched in ASCs compared to the paired SAT (Figure 3A,B). 

The ASC phenotype was further characterized in the immunological gate, evaluating the mesenchymal (CD90, CD73 and CD271) and the pericyte (CD146) surface marker expressions (Figure 3C). The finding that all antigens are similarly expressed in LT- and SAT-derived ASCs suggests the presence of quantitative and not qualitative alterations in the LT stem cell pool.

LT- and SAT-derived SVF cells were then compared by in vitro subculture, showing a similar morphology in human standard medium (h-SdM). In fact, adipose precursor cells isolated from both tissues display the typical fibroblast-like shape, appearing as flat and spindle cells (Figure 3D). In addition, we performed the proliferation analysis of the SVFs obtained from two MSL patients using both an ATP-based and a DNA-based assay. Cell cycle length was calculated as reported in the Materials and Methods section and expressed as the proliferation rate compared to that of SAT, which was arbitrarily set as 1. Notably, in both experiments, LT-derived SVF cells exhibit a doubling time significantly lower than SAT precursors, showing a proliferation rate about 30% higher than healthy AT (Figure 3E).

To further characterize MSL precursors that are mostly represented by ASCs (Figure 3B), we analyzed the gene expression profile of freshly isolated SVF cells to avoid bias due to culture conditions. Figure 3F shows that SAT- and LT-derived SVF cells display a similar expression of *PPARG2*, *LEPTIN*, *EVA1*, *CIDEA* and *ELOVL3*, as well as of *CD137*, a specific marker of beige precursors in animal models and in humans [26]. *UCP1* expression, which is lower in precursor cells (Figure 3G) compared to whole AT (Figure 1E), is near undetectable in both SAT- and LT-derived SFV cells, while it is clearly evident in Pheo-derived SVF cells.

### 2.4. LT-Derived ASCs Display a Great In Vitro White Adipogenic Potential

**Figure 4:**
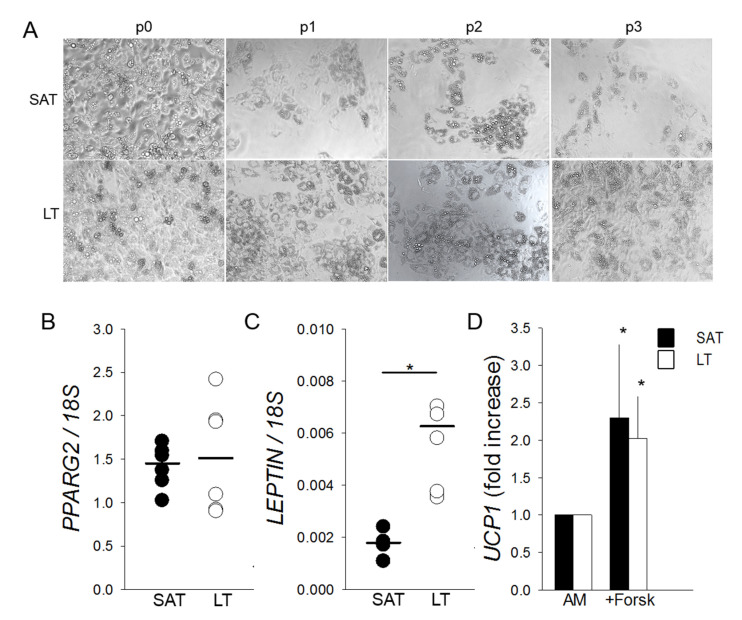
Adipogenic differentiation and browning of SVF cells derived from LT. (**A**) Representative photomicrographs (10× magnification) at the end of adipogenic differentiation of SAT and LT SVF cells at subsequent preliminary in vitro culture passages (p0–p3). (**B**,**C**) *PPARG2* and *LEPTIN* mRNA levels quantified in in vitro differentiated adipocytes obtained from SAT (black circles) and LT (white circles) of 3 MSL patients, normalized to the *18S* rRNA content (ratio of arbitrary units). Data are expressed as single values (circles) and medians (solid lines). * *p* < 0.05 SAT vs. LT, Mann-Whitney test. (**D**) *UCP1* expression upon stimulation of mature adipocytes from SAT (black bars) and LT (white bars) of 3 MSL patients with 10-µM Forskolin (+Forsk). *UCP1* levels are reported as fold increase with respect to control mature cells, untreated with Forskolin (AM). * *p* < 0.05 +Forsk vs. AM, Mann-Whitney test.

Freshly isolated SVF cells cultured in adipogenic conditions highlight that LT shows an adipogenic potential greater than SAT, which is maintained throughout the subculture passages (from p0 to p3) (Figure 4A) in all the analyzed patients (*n* = 6). Mature adipocytes, obtained from LT precursors and differentiated in vitro for 12 days, express at p0 a similar amount of *PPARG2* mRNA compared to SAT. Conversely, they contain a higher level of *LEPTIN* mRNA, suggesting both the presence of an increased number of mature white adipocytes and an upregulation of the most reliable white AT marker (Figure 4B,C). Moreover, SAT- and LT-derived adipocytes, differentiated for 12 days and stimulated to brown adipogenesis by treatment with the adenylyl cyclase inducer Forskolin, show a similar two-fold increase of *UCP1* induction (Figure 4D).

### 2.5. The Clonogenic and Adipogenic Potential of LT Are Higher Compared to Healthy SAT

**Figure 5:**
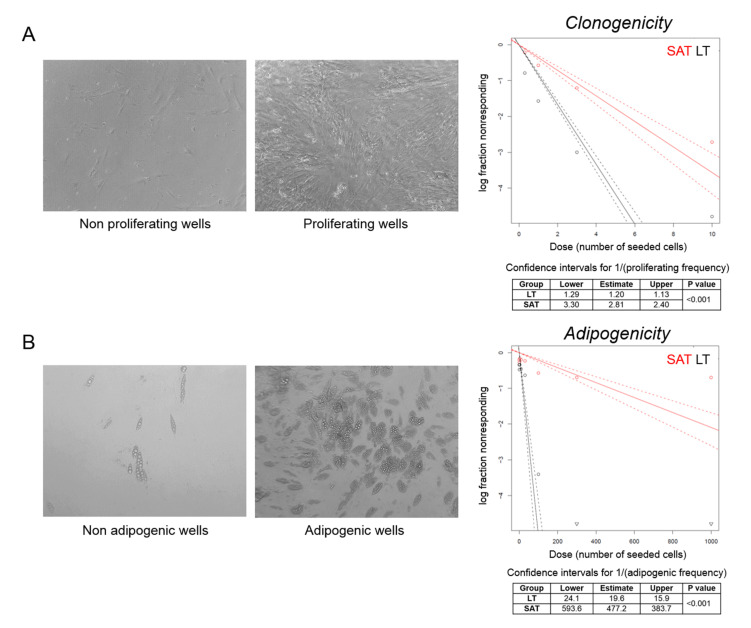
Clonal analysis of ASCs. (**A**,**B**) Representative photomicrographs (20× magnification) of nonproliferating vs. proliferating (cell confluence) and nonadipogenic vs. adipogenic (adipogenesis > 20%) wells counted to determine, respectively, the clonogenic and adipogenic potential of LT precursor cells derived from MSL patient 2891 I. The log fraction plots of nonproliferating (**A**) and nonadipogenic (**B**) wells, indicated as nonresponding, vs. the number of SAT (red lines) and LT SVF cells (black lines) seeded per well, are shown. Slopes of solid and dotted lines represent the log-active cell fraction and 95% confidence intervals, respectively. Confidence intervals for clonogenic and adipogenic frequency in the tested group (1/) and *p*-value were calculated by ELDA software (http://bioinf.wehi.edu.au/software/elda/).

We then performed a clonal analysis by limiting dilution assay of precursor cells obtained from LT and SAT collected during the first surgery of the MSL patient 2891. We seeded a different number of SVF cells per well and considered positive the wells where we observed confluent cells after three weeks of culturing by optical microscopy evaluation. Clonogenicity was estimated by plotting the log fraction of negative (nonresponding) wells against the number of seeded cells per well (Figure 5A). At high doses (1000, 300, 100 and 30 cells/well), all wells were positive, since the relative high number of seeded SVF cells from both LT and SAT were able to proliferate. Conversely, at low doses (10, 3, 1 and 0.3 cells/well), LT is characterized by a two or three-fold higher clonogenic activity compared to SAT (confidence intervals for 1/(proliferating frequency) SAT: 2.81, 2.40–3.30 vs. LT: 1.20, 1.13–1.29; *p* < 0.001) (Figure 5A).

To investigate the capacity to enter the adipogenic differentiation program, wells containing confluent cells were cultured in adipogenic differentiation medium for 12 days and analyzed by optical microscopy. Wells containing more than 20% of mature adipocytes were considered as positive (Figure 5B). Adipogenicity was evaluated by plotting the log fraction of negative (nonresponding) wells against the number of seeded cells per well (Figure 5B). The frequency of adipogenic precursor cells was significantly higher in LT than in SAT (confidence intervals for 1/(adipogenic frequency) SAT: 477.2, 383.7–593.6 vs. LT: 19.6, 15.9–24.1; *p* < 0.001) (Figure 5B), demonstrating that LT is significantly enriched with adipogenic precursor cells able to proliferate and differentiate under clonal culture conditions compared with SAT.

## 3. Discussion

The present study provides an extensive characterization of the LT from patients affected by MSL. Despite lipomatosis being a rare disease, we were able to enroll six unrelated patients with type I MSL, ensuring phenotype homogeneity. Importantly, each pathological sample was compared with a healthy SAT specimen obtained from the same MSL patient. Moreover, to validate our analysis, perirenal AT from patients affected by pheochromocytoma (Pheo) was used as a positive control of human browning in all our IHC and RNA expression experiments.

Preliminary studies considered LT as a BAT depot, defective of brown adipogenic differentiation, on the basis of its anatomical localization and UCP1 expression [3,9,13,27], despite the fact that the majority of LT histologies showed the presence of typical white adipocytes [28,29], and gene expression data were often contrasting [10,13,30,31]. Our histological analyses clearly showed that LT displays a typical white AT morphology with mature and univacuolated adipocytes, differently from the multivacuolated adipocytes of the positive browning controls. Moreover, our IHC and qPCR analysis provided the clear-cut evidence that LT does not stain for UCP1 protein and contains very low *UCP1* mRNA levels, similarly to the paired-control SAT. The gene expression analyses of other white/brown/beige markers in LT and LT-derived SVFs confirmed a white signature, which mainly overlaps that of healthy SAT.

**Figure 6:**
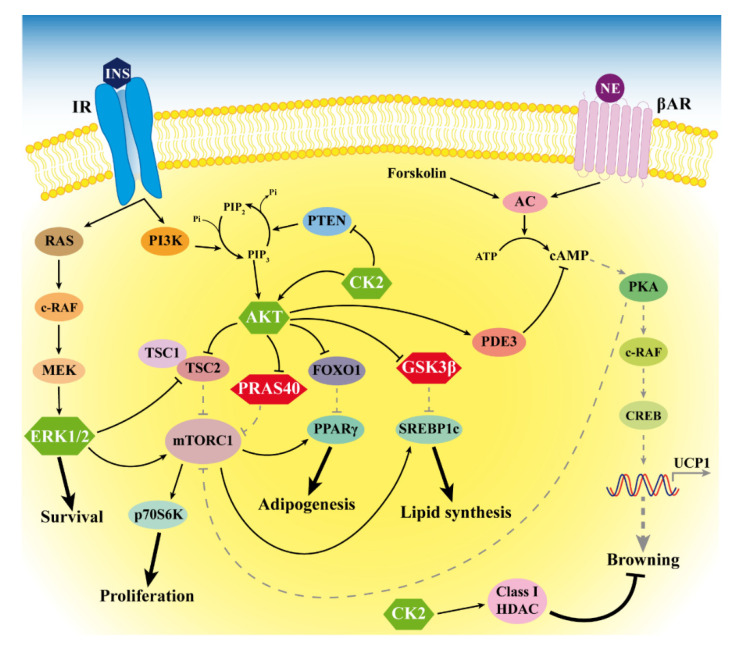
Signaling alterations in MSL LT. The figure depicts the anomalous hyperactivation of CK2, AKT and ERK1/2 (green hexagons) and the hyperphosphorylation of the AKT substrates PRAS40 and GSK3β (red hexagons) that we found in LT. Arrows and bar-headed lines indicate activation and inhibition, respectively. Black solid and grey dotted lines represent activated and inactivated pathways, respectively. HDAC: histone deacetylases.

For the first time, we showed the hyperactivation of the protein kinases AKT, CK2 and ERK1/2 in LT biopsies. The activity of these kinases is associated with the cell cycle, proliferation, survival and insulin signaling. In particular, the upregulation of AKT is consistent with previous data showing that mutation or knockout of the AKT-signaling inhibitor PTEN is associated with overgrowth syndromes involving AT depots [14,15,32]. Moreover, since AKT is also involved in insulin signaling [21], its activation suggests an increased tissue insulin sensitivity, which most likely contributes to LT expansion. A similar role could be played also by CK2, since we recently demonstrated that it is involved in adipocyte insulin sensitivity and whole-body glucose homeostasis. Moreover, we also found that CK2 is overexpressed and hyperactivated in WAT of patients with obesity and that weight loss is able to restore CK2 to a normal level [33]. The present study showing CK2 upregulation in LT of patients with MSL confirms that CK2 hyperactivation is a hallmark of AT expansion and increased adipogenic potential. CK2 was demonstrated to be specifically expressed in white mature adipocytes and to act as a negative modulator of browning by regulating class I histone deacetylases (HDACs) [34], suggesting that CK2 hyperactivation could be considered as a further WAT-specific feature of LT. An additional counteracting effect on adipocyte browning in LT might be exerted by the AKT hyperactivation that enhances the phosphodiesterase activity, leading to an increased cAMP degradation [35] (see Figure 6).

Our analysis of LT samples also demonstrated a consistent phosphorylation of the AKT substrates PRAS40 and GSK3β. PRAS40 phosphorylation is involved in protein synthesis and regulates fat tissue homeostasis by mTOR-signaling activation [23,36]. Consistently, Felthaus et al. recently showed that the inhibition of the mTOR pathway in LT-derived cells reduces their proliferation and differentiation capacity [37]. GSK3β phosphorylation regulates the glycogen and lipid metabolism [22,38], and the activation of these pathways could contribute to enhancing adipogenesis and supporting LT expansion. 

To investigate the mechanisms involved in the LT anomalous overgrowth characterized by the hyperactivation of several kinases involved in proliferation and adipogenic differentiation, we evaluated its stemness. 

Our analysis of LT SVFs highlighted the presence of a higher percentage of ASCs, with a similar immunophenotype (CD45−CD31−CD34+CD90+CD73+), compared to SAT SVFs. We found that LT precursor cells displayed an increased proliferation rate, as well as an enhanced in vitro differentiation capacity toward white adipocytes, as confirmed by the higher expression of *LEPTIN*, the master white adipokine.

Three different types of adipocytes have been characterized in animals and humans with different morphologies, specific metabolic functions and distinct embryological origins. White adipocytes contain a single, large lipid droplet for energy storage; brown adipocytes are multi-vacuolated, rich in mitochondria and burn lipids to produce heat and beige adipocytes display a white morphology but a brown function in response to environmental stimulation through UCP1 activation [39].

To reveal the possible presence of beige adipocytes in LT, we treated differentiated adipocytes with Forskolin [40] and obtained a similar upregulation of *UCP1* in LT- and SAT-derived cells, indicating the presence of only few beige cells in both tissues, as previously described in human AT [18,41].

Moreover, we quantified by clonal analysis a two or three-fold higher clonogenicity and 33-fold higher adipogenicity in LT compared to paired-control SAT. The increased adipogenic differentiation retained by LT-derived SVF cells could be explained by the increased amount of adipose-specific progenitors quantified ex vivo in the LT but, also, by their higher proliferation and clonogenic potential compared to SAT. In fact, these two last features could positively impact on the first step of adipogenic differentiation, characterized by mitotic expansion, and could improve the survival during the in vitro culturing. All these data demonstrated that LT preferentially expands by hyperplasia, displaying an increased number of ASCs and increased proliferation and clonogenic potential. The hyperplastic, rather than a hypertrophic, growth of LT, suggested also by Prantl et al. [42], has been further confirmed by our histological finding that the adipocyte cell size was smaller in LT than in healthy SAT. Our results suggest that this hyperplastic growth could be sustained by the increased activity of ERK1/2 and AKT we found in LT.

Moreover, we clearly showed that LT of MSL patients is characterized by an increased capacity to differentiate into white mature adipocytes, probably through the activation of several intersecting pathways with the involvement of AKT and CK2 and independently from *PPARG2* upregulation.

In MSL patients, the proliferative advantage seems strictly coupled with an increased adipogenic differentiation potential, which could protect the tissue against a tumoral deregulation, allowing only a local “benign” LT expansion and identifying MSL as a non-neoplastic disease.

Our study has some limitations. In particular, we were able to enroll only five male and one female patients with MSL due to the rarity of the disease and the prevalence in men. We showed the morphology of LT and the expression of some white and brown/beige specific genes, but we did not investigate miRNA involvement in the increased LT white adipogenesis. Our results suggest the role of different kinases associated to the insulin pathway in the LT expansion, but future studies will be necessary to deeply define the molecular mechanisms at the basis of the alterations we observed. In Figure 6, we summarize our results, and we hypothesize that LT signaling alterations occur in MSL patients, as suggested by our study and previous reports. 

The anomalous activation of CK2, AKT and ERK1/2 in LT suggests that a downregulation of these kinases might represent a promising pharmacological tool to prevent MSL lipoma expansion and relapse. For these reasons, the CK2 inhibitor CX-4945, currently used in oncological clinical trials (NCT02128282), as well as the ERK1/2-inhibitors ulixertinib (BVD-523) or LTT462 (NCT01781429 and NCT02711345, respectively), could be considered as interesting drugs targeting LT to prevent recurrent surgery.

## 4. Materials and Methods

### 4.1. Clinical Patient Characterization and Tissue Collection

Six patients described in Table 1A–C (ID 2884, 2891, 3002, 3030, 5091 and 5937), affected by type I MSL, who underwent lipectomy for clinical indication at the Clinic of Plastic and Reconstructive Surgery of Padua Hospital, were enrolled in the present study, approved by the Padua Ethical Committee for Clinical Research, after written informed consent (10 April 2014 Prot. N° 2658P).

**Figure 7:**
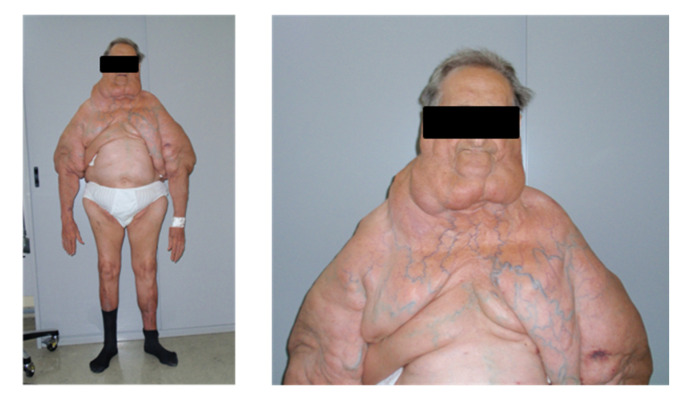
Features of type I MSL. Representative pictures of a patient affected by type I MSL. LT around the neck forms the so-called Madelung collar. LT involves the proximal arms and the upper trunk, sparing the abdomen and legs.

LT and a paired biopsy of SAT in the MSL-spared site were collected from the same patient during surgical lipectomy; in two patients (2891 and 3002), samples of both LT and SAT were collected twice during two consecutive interventions, indicated by ordinal numbers (I and II). LT samples derived from the neck or cervical anatomical region, named Madelung collar (patients 2884, 2891 I and II, 3002 I and 5091), and from upper arms (patients 3002 I and II, 3030, 5091 and 5937), whereas SAT mostly derived from the abdominal, lumbar (3002 I) or lower limb (3002 II) regions. The range of age was 49–70 years, and at least one year of alcohol abstinence was requested as inclusion criteria for the enrollment. For each patient, at the time of each surgery, anthropometric parameters (weight and height) were collected, and BMI was computed (weight in kilograms/(height in meters)^2^) (Table 1A). Blood biochemical analyses were performed after an 8-h fast. Biochemical measurements (Table 1C) were performed using diagnostic kits standardized according to the World Health Organization First International Reference Standard. A photographic documentation was performed in order to assess the sites and extension of the disease (Figure 7).

The M/F ratio (5/1) of our cohort reflects the higher prevalence of type I MSL in males; two patients (ID 3030 and 5937) were affected by type 2 diabetes mellitus (T2D), and 5 patients had a history of alcohol abuse; BMI ranged from 22.5 and 33.8 kg/m^2^: one subject (2884) resulted normal weight and 3 overweight (2891, 3030 and 5091), while patient 3002 was overweight during the first intervention (3002 I) and resulted in being affected by I degree obesity at the time of the second surgery (3002 II), as well as patient 5937. Clinical data and the sites of LT and SAT collection are summarized in (A) in Table 1. Perirenal AT was harvested from 4 patients affected by pheochromocytoma during adrenalectomy, following surgery standard written informed consent.

### 4.2. Histological and IHC Analysis

LT and SAT samples were fixed in 4% buffered formaldehyde (Diapath S.p.A, Bergamo, Italy), paraffin-embedded, cut into 5-μm-thick sections and stained with standard H&E. Tissue sections were observed under a Leica DM LB2 light microscope, at 20× magnification, and at least 20 fields for each specimen were evaluated to determine adipocyte morphology. Digital images were captured with a Leica DFGC450 digital camera in at least 10 fields per specimen to manually measure the adipocyte size in at least 160 random adipocytes, using LAS Software (Leica Microsystems Inc., Deerfield, IL, USA). IHC was performed using a Bond-maX automated IHC stainer (Leica, Newcastle Upon Tyne, UK), applying the standard protocol. In brief, sections were pretreated using heat-mediated antigen retrieval with sodium citrate buffer (pH 6) for 30 min, then incubated with rabbit polyclonal anti-UCP1 antibody (1:250 dilution, catalog ab10983, Abcam, Cambridge, UK) for 15 min at room temperature and detected using a Dako-labeled streptavidin biotin-horseradish peroxidase kit. Hematoxylin was used for counterstaining. Sections were then dehydrated, cleared, mounted and observed with a Leica DM LB2 light microscope.

### 4.3. RNA Extraction and Real-Time PCR

Total RNA was extracted using RNeasy Lipid or Mini Kits (Qiagen Inc., Valencia, CA, USA) for tissue and cell culture samples, respectively, following the supplier’s instructions. RNA content was quantified using NanoDrop technology (Fisher Scientific SAS, Illkirch Cedex, France) and quality checked using an Agilent 2100 Bioanalyzer (Agilent Technologies, Santa Clara, CA, USA). RNA samples were treated with DNase Treatment and Removal Reagents (Thermo Fisher, Waltham, MA, USA) and reverse-transcribed for 1 h at 37 °C with 150-ng random primers, 0.5-mM dNTPs, 20 units of RNAsin Ribonuclease Inhibitor and 200 units of M-MLV RT (Promega, Madison, WI, USA). Primer sequences and amplification conditions are reported in Table 2. Real-time PCR was carried out with Platinum^®^ SYBR^®^ Green qPCR SuperMix-UDG (Thermo Fisher) on a DNA Engine Opticon^TM^ 2 Continuous Fluorescence Detection System (MJ Research, Waltham, MA, USA). All experiments were performed in duplicate. Samples (5 ng of cDNA) were normalized by *18S* rRNA content and reported as an arbitrary unit (a.u.) ratio or as a fold increase/decrease with respect to the control.

### 4.4. Protein Extraction

Tissues were minced; covered by a cold lysis buffer containing 50-mM HEPES, pH 7.5, 150-mM NaCl, 10% glycerol, 5-mM Triton-X-100 and protease/phosphatase inhibitor cocktails (Calbiochem, Darmstadt, Germany and Sigma-Aldrich, St. Louis, MO, USA) and homogenized with Dounce homogenizer for 20 min. Tissue extracts were centrifugated (16,000× *g*, 15 min), and protein concentration was determined in the supernatants by the colorimetric Bradford method.

### 4.5. CK2 Kinase Activity Assay

CK2 kinase activity was performed as described previously [43]. In brief, protein lysates (5 µg) were incubated for 10 min at 30 °C in 25 μL of a phosphorylation medium containing 50-mM Tris-HCl (pH 7.5), 100-mM NaCl, 12-mM MgCl_2_, 400-μM synthetic peptide substrate RRRADDSDDDDD (kindly provided by Dr. O. Marin—University of Padua, Italy) and 20-µM (γ^33^P)ATP (1000–3000 cpm/pmol; PerkinElmer, Waltham, MA, USA). Assays were stopped by absorption onto phosphocellulose p81 filters (PerkinElmer). Filters were washed four times in 75-mM phosphoric acid and analyzed by a Scintillation Counter (PerkinElmer). CK2 activity, which is proportional to radioactivity, was expressed as cpm of ^33^Pi transferred to the peptide substrate.

### 4.6. Western Blot

Protein lysates were separated by 11% SDS-PAGE and blotted on Immobilon-P PVDF membranes (Sigma-Aldrich), following the manufacturer’s instructions and using TE 22 Mini Tank transfer unit (GE Healthcare, Waukesha, WI, USA). Membranes were incubated overnight with the following primary antibodies: CK2α/CK2α′ (MCA3031Z; Bio-Rad, Hercules, CA, USA); anti-CK2β (catalog ab76025) from Abcam (Cambridge, UK); anti-p-Akt (Ser473) (catalog #4060), anti-p-PRAS40 (Thr246) (catalog #13175), anti-p-GSK3β (Ser9) (catalog #5558), anti-GSK3β (catalog #9336), anti-p-ERK1/2 (Thr202/Tyr204) (catalog #4370) and anti-ERK1/2 (catalog #4695) from Cell Signaling Technology (Danvers, MA, USA); anti-Akt1/2/3 (catalog sc-8312) from Santa Cruz Biotechnology (Santa Cruz, CA, USA) and anti-β-actin (catalog A5441) from Sigma-Aldrich (Burlington, MA, USA). After removal of primary antibodies and washing with Tris-buffered saline (TBS), membranes were incubated with the secondary antibodies towards rabbit and mouse IgG, conjugated to horseradish peroxidase (PerkinElmer). A signal was developed using an enhanced chemiluminescent detection system ECL (Amersham Biosciences, Little Chalfont, UK). Immunostained bands were quantified by means of a Kodak Image Station 4000MM-PRO and analyzed with Carestream Molecular Imaging software (New Haven, CT, USA).

### 4.7. Stromal Vascular Fraction (SVF) Isolation, Morphological Analysis and Adipogenic Differentiation

SVF cells from LT and SAT biopsies were freshly obtained, as previously described [44]. For morphological observations, 0.4 × 10^6^ SVF cells/well (24-well plates) were seeded in duplicates in h-SdM and observed after 2 days (p0) with a Leica DM IL LED inverted microscope equipped with a camera. For subsequent culture passages (p1–p3), cells were detached with 0.025% trypsin/EDTA (Thermo Fisher), and 0.4 × 10^6^ cells/well (24-well plates) were than seeded for further optical microscopy observation after 48 h. For in vitro adipogenic differentiation, 0.5 × 10^6^ cells/well (24-well plates) from the SVF at different culture passages (p0–p3) were seeded in h-SdM. At cell confluence, the medium was replaced with human adipogenic medium (h-AdM) and replaced every 3 days with fresh h-AdM without 3-Isobutyl-1-methylxanthine (IBMX) and rosiglitazone [33]. At day 12, representative images of in vitro differentiated cells were taken with a Leica DM IL LED inverted microscope, and then, cell cultures were lysed using RLT buffer (Qiagen Inc.) for RNA extraction. To stimulate browning, at day 12 of differentiation, insulin, dexamethasone and T3 were removed, and cells were washed out 48 h and treated 4 h with 10-µM Forskolin (Sigma-Aldrich) before cell lysis.

### 4.8. Flow Cytometric Analysis

SVF cells, from paired samples of LT and SAT of MSL patients, were freshly isolated for an ex vivo multiparametric flow cytometry analysis to quantify and characterize the ASCs present in the SVF, avoiding modifications due to plastic adhesion and culture conditions. Cells (1 × 10^5^) were washed with cold FACS buffer (2% bovine serum albumin (BSA) in phosphate-buffered saline (PBS)), collected by centrifugation (350× *g*, 10 min) and simultaneously incubated in the dark for 10 min at room temperature with the following monoclonal mouse anti-human fluorochrome-conjugated antibodies against the indicated surface markers in different combinations, as reported in Table 3: CD31-FITC and CD31-PE, CD45-FITC, CD34-PerCP-Cy5.5, CD73-APC, CD90-PE, CD271-APC and CD146-PE (BD Biosciences, San Jose, CA, USA). Labeled cells were acquired and analyzed by a FACSCanto^TM^ Flow Cytometer (BD Biosciences) using the BD FACSDiva™ software, as detailed previously by Belligoli et al. [45], to quantify and characterize the mature endothelial cells (CD45−CD31+CD34−), endothelial progenitor cells (CD45−CD31+CD34+) and ASCs (CD45−CD31−CD34+) [46,47].

### 4.9. Proliferation Assays

Freshly isolated SVF cells/well from paired LT and SAT samples (1 × 10^4^) were seeded in 96-well plates in triplicates in h-SdM, and the proliferation capacity was determined by two different methods. At time 0 (t0) and 48 h after seeding, the number of viable cells was determined using the CellTiter-Glo^®^ Luminescent Cell Viability Assay (Promega) according to the manufacturer’s protocol. The day of the assay, the plates were equilibrated to room temperature for 30 min, and 1:1 volume of CellTiter-Glo^®^ reagent, equivalent to the amount of medium where the cells were plated, was added to each well and mixed for 2 min to induce cell lysis. After 10 min of incubation, emitted luminescence, proportional to the content of ATP, was detected using a Victor3^TM^ microplate reader (PerkinElmer). At t0 and 48 h after cell seeding, the cell number was determined using the CyQUANT^®^ Direct Cell Proliferation Assay Kit (Thermo Fisher), according to the manufacturer’s instructions. This DNA-based proliferation assay utilized a cell-permeant DNA-binding fluorescent dye. At the indicated time point, an equal volume of 2× detection reagent was added to each well, and, after 60 min of incubation at 37 °C, the fluorescence was read at 480/535 nm using a FluoStar Optima microplate reader (BMG Labtech, Otterberg, Germany).

In both methods, the doubling time (g) during the logarithmic growth phase was calculated with the following equation: g = Th × log2/log(N_Th_/N_0h_), where Th indicates the time of culture in hours, N_Th_ the estimated number of cells after 48 h of culture and N_0h_ the number of cells at t0. Data of 2 independent experiments performed with the 2 different methods were reported as the relative proliferation rate of LT-derived SVF cells with respect to SAT-derived cells of the same patients, arbitrarily set as 1.

### 4.10. Limiting Dilution Analysis

SVF cells derived from LT and SAT were serially diluted (1:3); seeded in 96-well plates at different concentrations (1000, 300, 100, 30, 10, 3, 1 and 0.3 cells/well) and grown in h-SdM. Sixty replicate wells were generated for each condition. After 3 weeks, the wells were scored for cell proliferation by observation under a Leica DM IL LED inverted microscope, counting those with 100% of cell confluence (responding wells). The number of nonproliferating (nonresponding) wells were statistically analyzed.

To evaluate the adipogenic potential, proliferating wells were switched to adipogenic medium, as described above, and then, differentiated wells were scored for the presence of adipocytes, estimating the percentage of mature adipocytes by double-blind observation with a Leica DM IL LED inverted microscope upon standard Oil Red O staining [45]. The number of adipogenic wells (mature adipocytes ≥ 20% of the total cells; responding wells) were counted, and nonadipogenic (nonresponding) wells were statistically analyzed.

### 4.11. Statistical Analysis

Results are presented as mean ± SD or as median, 25th and 75th or 5th and 95th percentiles. Variables were tested for normality using the Shapiro-Wilk test, and statistical analysis was performed using the unpaired Student’s *t* (two-tailed)-test for normal distributed variables or Mann-Whitney nonparametric test for skewed data. All the *p*-values were two-sided, and differences were considered statistically significant at *p*-values lower than 0.05. Statistical analysis was performed using SigmaPlot 13.0 Systat Software, Inc. (San Jose, CA, USA). Limiting dilution analysis data were elaborated using a web application made available by the Walter and Eliza Hall Institute of Medical Research, Parkville Victoria, Australia (http://bioinf.wehi.edu.au/software/elda/) based on the single-hit Poisson model using a generalized linear model [48].

## Figures and Tables

**Table 1:** Characterization of patients affected by multiple symmetric lipomatosis (MSL) at the time of surgery. (**A**) Demographic, anthropometric, metabolic characteristics, (**B**) smoking and alcohol usage history and (**C**) biochemical parameters of MSL patients.

**(A)**							
**ID**	**Gender**	**Age**	**MSL Type**	**LT Site**	**SAT**	**T2D**	**BMI**
2884	M	58	I	Neck	Abdominal	no	22.5
2891 I	M	62	I	Neck	Abdominal	no	26.6
2891 II	M	63	I	Neck	Abdominal	no	26.5
3002 I	M	64	I	Neck and upper arm	Lumbar region	no	29.7
3002 II	M	67	I	Upper arm	Lower limb	no	33
3030	M	49	I	Upper arm	Abdominal	yes	29.4
5091	M	70	I	Neck and upper arm	Abdominal	no	28.3
5937	F	63	I	Upper arm	Abdominal	yes	33.8
**(B)**							
**ID**	**Alcohol**		**Alcohol Amount (L/Day)**		**Smoking Status**	**Smoking Frequency** **(Cigarettes/Day)**	
2884	yes		-		never	-	
2891	yes		2 (red wine)		current	15	
3002	yes		-		never	-	
3030	no		-		current	20	
5091	yes		1 (red wine)		previous	-	
5937	yes		-		never	-	
**(C)**							
**ID**	**FPG** **(mmol/L)**	**Total Chol** **(mg/dL)**	**LDL-Chol** **(mg/dL)**	**HDL-Chol** **(mg/dL)**	**Triglycerides** **(mg/dL)**	**ALAT** **(UI/L)**	**GGT** **(UI/L)**
2884	4.9	200	139	41	150	18	-
2891	4.2	148	61	52	170	-	47
3002	5.9	152	109	24	95	23	45
3030	8.5	191	129	46	80	26	22
5091	5.4	151	39	92	105	23	79
5937	6.3	183	109	36	189	26	32

ID, identification number of each patient; I and II, intervention order; M, male; F, female; LT, lipomatous tissue; SAT, subcutaneous adipose tissue; T2D, type 2 diabetes mellitus; BMI: body mass index; FPG, fasting plasma glucose; LDL, low-density lipoprotein; Chol, cholesterol; HDL, high-density lipoprotein; ALAT, alanine aminotransferase and GGT, gamma-glutamyl transpeptidase.

**Table 2:** Primer sequences and real-time PCR conditions.

Gene	Forward (5′-3′) Reverse (5′-3′)	Annealing (°C)	Primers (F/R nM)	Amplicon (bp)
*PPARG2*	ACCCAGAAAGCGATTCCTTCA AGTGGTCTTCCATTACGGAGAGATC	60	900/900	87
*LEPTIN*	GTGCGGATTCTTGTGGCTTT GGAATGAAGTCCAAACCGGTG	63	100/100	174
*UCP1*	CTACGACACGGTCCAGGAGT AGTGGCAGTATTCATTGGGC	60	300/300	110
*CIDEA*	ACGTGAAGGCCACCATGTATGA TGCCCAGATAGATGAGAAACTGTCC	62	300/300	141
*ELOVL3*	CCTTGCAATCTTCAGTATCCTGG GATGAAGTTGATGAAGCACACG	60	300/300	146
*EVA1*	CAGTTCGACGACAATGGGACAT AGAGAAGCGTACAGTGTGCACGA	60	300/300	108
*CD137*	CGACCCTGGACAAACTGTTCTTT AAGGAGATGATCTGCGGAGAGTGT	63	300/300	170
*18S*	CGGCTACCACATCCAAGGAA GCTGGAATTACCGCGGCT	60	100/100	186

*PPARG2*, peroxisome proliferator activated receptor gamma isoform 2; *UCP1*, uncoupling protein 1; *CIDEA*, cell death-inducing DFFA-like effector A; *ELOVL3*, elongation of very long chain fatty acid-like 3; *EVA1*, epithelial V-like antigen 1; *CD137*, tumor necrosis factor receptor superfamily, member 9 and 18S, 18S ribosomal RNA.

**Table 3:** Antibody flow cytometry panel for the immunophenotyping of SVF cells. SVF cells, isolated from LT and SAT of patients affected by MSL, were incubated with various combinations of monoclonal mouse anti-human fluorochrome conjugated antibodies specific for the different antigens in order to perform a multiparametric flow cytometric analysis. Isotype-matched FITC-, PE-, PerCy5.5- and APC-IgG1 monoclonal antibodies were used as negative controls. Unstained samples were used for autofluorescence control.

Samples	FITC	PE	perCP-Cy5.5	APC
Unstained	-	-	-	-
Negative	IgG1	IgG1	IgG1	IgG1
Sample 1	CD45	CD31	CD34	-
Sample 2	CD45 CD31	CD90	CD34	CD73
Sample 3	CD45 CD31	CD146	CD34	CD271

## Abbreviations

AKT	Protein kinase B
ALAT	Alanine aminotransferase
ASC	Adipose Stem Cell
AT	Adipose Tissue
BAT	Brown Adipose Tissue
BMI	Body Mass Index
BSA	Bovine Serum Albumin
CAPSL	Calcyphosin-like
CIDEA	Cell death inducing DFFA like effector A
CK2	Casein kinase 2
EDTA	Ethylenediaminetetraacetic acid
ELOVL3	Elongation of very long chain fatty acid-like 3
ERK1/2	Extracellular signal-regulated kinase 1/2
EVA1	Epithelial V-like antigen 1
GGT	Gamma-glutamyl transpeptidase
GSK3β	Glycogen synthase kinase-3 beta
h-AdM	Human Adipogenic Medium
H&E	Hematoxylin and Eosin
h-SdM	Human Standard Medium
IBMX	3-Isobutyl-1-methylxanthine
IHC	Immunohistochemistry
LT	Lipomatous Tissue
MFNT2	Mitofusin 2
MSL	Multiple Symmetric Lipomatosis
mTOR	Mammalian target of rapamycin
PBS	Phosphate-buffered saline
PCR	Polymerase Chain reaction
PPARG2	Peroxisome proliferator-activated receptor gamma
PRAS40	Proline-rich AKT substrate
PTEN	Phosphatase and tensin homolog
RhoA	Ras Homolog Family Member A
ROCK	Rho-associated protein kinase
SAT	Subcutaneous Adipose Tissue
SVF	Stromal Vascular Fraction
TBS	Tris-buffered saline
T2D	Type 2 diabetes mellitus
UCP1	Uncoupling Protein 1
WAT	White Adipose Tissue
